# The Microbial Diversity and Traceability Analysis of Raw Milk from Buffalo Farms at Different Management Ranks in Guangxi Province

**DOI:** 10.3390/foods13244080

**Published:** 2024-12-17

**Authors:** Wenhao Miao, Dong Wang, Ling Li, Enghuan Hau, Jiaping Zhang, Zongce Shi, Li Huang, Qingkun Zeng, Kuiqing Cui

**Affiliations:** 1 State Key Laboratory for Conservation and Utilization of Subtropical Agro-Bioresources, Guangxi University, Nanning 530004, China; percenatania@gmail.com (W.M.); wangdong20201214@163.com (D.W.); lling2010@163.com (L.L.); 2 Guangxi Zhuang Autonomous Region Buffalo Milk Quality and Safety Control Technology Engineering Research Center, Guangxi Buffalo Research Institute, Chinese Academy of Agricultural Sciences, Nanning 530001, China; enghuan_90@yahoo.com (E.H.); zhangjiaping1997@163.com (J.Z.); 17784454557@163.com (Z.S.); huangli00206@163.com (L.H.); 3 Guangdong Provincial Key Laboratory of Animal Molecular Design and Precise Breeding, School of Life Science and Engineering, Foshan University, Foshan 528225, China

**Keywords:** raw buffalo milk, farming environments, microbiota, 16S rRNA gene sequencing

## Abstract

Farm management has a significant impact on microbial composition and may affect the quality of raw buffalo milk. This study involved a diversity analysis and traceability of the microbial communities in raw buffalo milk from three buffalo farms at different management ranks in Guangxi Province, China. The microbial composition of the raw buffalo milk and its environmental sources were investigated using 16S rRNA gene sequencing and bioinformatics analysis. The results demonstrated that different management ranks significantly influenced microbial composition in milk, with the primary sources of contamination varying across farms. The *env.OPS_17* was the predominant differential bacterium in farm rank A, whereas *Enterobacteriaceae*, *Aerococcaceae*, and *Planococcaceae* were dominant in farm rank B. The Fast Expectation–Maximization for Microbial Source Tracking model revealed that while the sources of microbial contamination varied across farms at different management ranks, the teat and teat liner consistently emerged as the primary sources of microbial contamination in raw buffalo milk. This study provides important insights into how different farm management ranks affect the microbial composition of raw buffalo milk, highlighting the importance of improved management practices during milk production, particularly in cleaning the milking equipment and farm environment, as these are key factors in ensuring the quality and safety of raw buffalo milk.

## 1. Introduction

Buffalo is the second-largest source of milk worldwide, and its milk production has been steadily increasing, to 143.6 billion liters by 2022, accounting for approximately 19% of the total milk output [1]. Buffalo milk and its dairy products hold significant importance in the dietary patterns of certain Asian and European countries [2]. Their bioactive peptides with antihypertensive, antioxidant, antidiabetic, and antimicrobial properties are widely acclaimed by local populations [3]. Buffalo milk has high fat and protein contents, so it serves as a key ingredient in many high-value dairy products, including cheese and yogurt. The unique processing methods for buffalo milk, such as milk collection, transportation, and cheese production, require stringent hygienic standards, especially since some cheese production processes do not involve high-temperature pasteurization [4]. Hence, microbial control in raw buffalo milk is of critical importance.

Farm management plays a crucial role in determining the microbiological quality of buffalo milk, as highlighted in a previous study, where environmental contaminants were often identified as carriers of microbial transmission [5]. The environment of a dairy farm is inherently dynamic, and the microbial community is an important part of this ecosystem [6]. This complexity poses a great challenge, especially in large-scale buffalo farming, as milk is susceptible to microbial contamination from different sources throughout the production process [7]. Microbial contamination during and after milking can originate from various environmental sources, including feed, feces, bedding, and soil. These contaminants adhere to the udder surface and are subsequently transferred into the milk [8]. Therefore, improving farm management practices can substantially reduce the impact of environmental microorganisms on the microbial profile of raw milk.

Previous research on raw milk and dairy products has widely employed both culture-dependent and culture-independent approaches to analyze microbial compositions [9]. Isolating and culturing microorganisms under specific laboratory conditions helps researchers to understand their survival mechanisms, metabolic products, and interactions with other microbes. Furthermore, advances in molecular techniques, especially high-throughput DNA sequencing, have enabled a deeper understanding of the microbial communities in raw milk and their potential sources of contamination [10]. For instance, high-throughput DNA sequencing can identify microorganisms that are viable but non-culturable, detect extremely rare microbes in the community, and accurately determine the relative abundance of various microbes in the environment [11].

Microbial source tracking (MST) methods have been developed to track environmental microbial contaminants, often using molecular markers like crAssphage [12]. However, a notable exception is Source Tracker, which is currently the most widely used microbial source tracking method [13]. Unlike previous approaches, Source Tracker employs a Bayesian framework to estimate the proportion of contaminants in a given community by leveraging the structure of specific communities and measuring the similarity between the sink community and potential source environments [14]. Despite its widespread use, Source Tracker has notable limitations, including long computational times and reduced accuracy when estimating contributions from unknown sources [15]. The recent Fast Expectation-Maximization for Microbial Source Tracking (FEAST) method, based on the principles of Source Tracker, improves computational efficiency and accuracy using an expectation-maximization approach, in which it quantifies source contributions from microbial data and offers reliable insights for tracking the sources of microbial and resistance genes [16]. It helps identify key contamination factors in milk, such as feed, water, or equipment, and assesses the impact of management practices.

Our previous research confirmed significant differences in the microbiota of raw buffalo milk among farms with different milking methods in Guangxi Province [17]. To explore the origins of these differences, we selected three representative farms at varying management ranks to collect environmental and raw buffalo milk microbiota samples, aiming to identify the risk points and specific microbial groups requiring attention during farm operations. This study provides valuable insights into how the farming environment influences milk microbiota, helping buffalo farms enhance management practices to minimize bacterial contamination during milking and processing, thereby improving milk quality. Despite the importance of this issue, limited research in China has investigated microbiota diversity in buffalo farm environments using 16S rRNA sequencing, and no studies have specifically examined microbiota profiles across buffalo farms in relation to milk. By employing culture-independent methods, this study advances the understanding of contamination sources in raw buffalo milk while offering practical guidance for pasture management and contamination prevention.

## 2. Materials and Methods

### 2.1. Sample Collection

Canned buffalo milk and risk-related production processes were studied at three large-scale buffalo farms in Guangxi Province in January 2023. Based on the operational conditions of different farms, a total of 87 samples were collected for raw buffalo milk microbiological traceability. A 0.09% sodium chloride (NaCl) solution was used as the sterile medium for all relevant procedures. Milk samples were collected directly from milk cans at the buffalo farms using sterile stainless steel sampling spoons (VWR, Radnor, PA, USA), which were sterilized in an autoclave (Tomy SX-500, Tomy Digital Biology Co., Tokyo, Japan) before use and handled with gloves disinfected with 75% ethanol. Drinking water samples were collected in equal volumes from drinking troughs in each barn using sterile 50 mL conical tubes and mixed thoroughly in a sterile container to ensure homogeneity. Pond water was sampled using the random sampling method to ensure representativeness; equal volumes were collected in pre-sterilized sampling bottles, thoroughly mixed, and sealed immediately after collection to prevent contamination. Muslin cloth samples were prepared by soaking the gauze used for filtering milk in sterile NaCl solution, re-suspending it, and homogenizing it using a vortex mixer handled with sterile forceps to avoid contamination. Teat liner samples were obtained by swabbing each liner along its inner walls with sterile swabs pre-soaked in the NaCl solution, which were then re-suspended in 20 mL of sterile NaCl solution in sterile centrifuge tubes. For the inner surfaces of milking unit tubes, thorough swabbing was performed using sterile swabs pre-soaked in the NaCl solution, and the swabs were re-suspended in 20 mL of sterile NaCl solution in 50 mL centrifuge tubes. Teat dip cup samples were collected by swabbing the inner walls of each dip cup with sterile swabs, which were then re-suspended in 20 mL of sterile NaCl solution and stored in sterile 50 mL centrifuge tubes. Teat samples were obtained by swabbing each buffalo teat with sterile swabs, which were re-suspended in 20 mL of sterile NaCl solution in sterile centrifuge tubes. Milk canister and milk transfer bucket samples were collected by swabbing the bottoms of each container with sterile swabs, which were then re-suspended in 20 mL of sterile NaCl solution in sterile centrifuge tubes. Feed samples were collected in equal amounts from each barn using sterile sampling spoons, thoroughly mixed in sterile bags, and sealed to maintain sterility. Fecal samples were collected in equal amounts from the manure pits in each barn using sterile sampling spoons, thoroughly mixed, and stored in sterile bags. For all sample types, sterile instruments and containers were used, and sterility was ensured by regular sanitization with 75% ethanol and careful handling. All environmental and milk samples were transported on dry ice and stored in a laboratory freezer at −80 °C until further analysis.

### 2.2. DNA Extraction

Metagenomic DNA of microorganisms from raw buffalo milk, feed, feces, teat liners, water, teats, milk canisters, milk transfer buckets, pools, muslin cloths, and teat dip cups was extracted using the PowerSoil^®^ DNA Isolation Kit (Mo Bio Laboratories, Carlsbad, CA, USA) following the manufacturer’s standard protocol.

### 2.3. 16S rRNA Gene Sequencing

The V3-V4 region of the 16S rRNA gene was amplified using specific primers (338F: 5′-ACTCCTACGGGAGGCAGCA-3′; 806R: 5′-GGACTACHVGGGTWTCTAAT-3′). PCR reactions were conducted on a Bio-Rad T100 gradient PCR instrument (Bio-Rad, Hercules, CA, USA) using KOD polymerase (Toyobo, Osaka, Japan). The cycling conditions included an initial denaturation at 98 °C for 2 min, followed by 30 cycles of denaturation at 98 °C for 30 s, annealing at 50 °C for 30 s, and extension at 72 °C for 60 s, with a final extension at 72 °C for 5 min.

The resulting PCR products were quantified by electrophoresis, pooled in a 1:1 mass ratio, and purified using OMEGA DNA purification columns (Omega Bio-Tek, Norcross, GA, USA). Subsequently, the purified PCR products were separated on a 1.8% agarose gel at 120 V for 40 min. Target fragments were excised from the gel, and the purified products were recovered using a Qiagen Gel Extraction Kit (Qiagen, Hilden, Germany).

Following purification, the raw sequencing data underwent quality filtering using Trimmomatic (version 0.33), with primer sequences identified and removed using Cutadapt (version 1.9.1) [18,19]. Paired-end reads were merged, and chimera sequences were removed using USEARCH (version 10) with the UCHIME algorithm (version 8.1) [20,21], resulting in high-quality sequences for downstream analysis. Denoising was performed using the DADA2 method in QIIME2 software (version 2020.6.0) to obtain ASVs, applying a threshold of 0.005% of the total sequencing reads for filtering [22]. Taxonomic annotation was conducted in QIIME2 using the Silva database. Alpha diversity was calculated in QIIME2 and expressed as “mean ± standard deviation”. LEfSe (Linear Discriminant Analysis of Effect Size) software (version 1.0) was used to perform LEfSe analysis (LDA score ≥ 2) [23]. In addition, PICRUSt2 software (version 2.1.2-b) was used for functional annotation analysis to study the functions of communities in the sample and identify the different functions of communities in different groups [24]. FEAST analysis was performed in R (v3.6.3), with raw buffalo milk samples as the target samples (sink) and environmental samples (including feces, feed, teat liners, etc.) as the sources [16]. The FEAST tracing model was used to explore the microbial community sources in the target samples, predicting the composition of sinks based on the community structure of the sources and sinks. All analyses were conducted using the default parameters for FEAST. Statistical analyses were conducted using IBM SPSS Statistics (version 25). The data were initially tested for normality using the Shapiro–Wilk test to ensure suitability for parametric tests. Comparisons between groups were performed using Student’s *t*-test for pairwise comparisons and one-way Analysis of Variance (ANOVA) for multiple group comparisons. When ANOVA indicated significant differences (*p* < 0.05), post hoc analyses, such as Tukey’s Honestly Significant Difference (HSD) test, were employed to identify specific group differences. All data were reported as mean ± standard deviation unless otherwise specified. Bar charts for bacterial community structure and pie charts for community contributions were generated in GraphPad Prism (version 9.5.0). To evaluate the complexity of community composition and compare the differences between groups, the weighted and unweighted UniFrac distances in QIIME2 (version 2020.6.0)were used to calculate beta diversity [22]. Beta diversity evaluations of microbiome differences between samples were visualized using Principal Coordinate Analysis (PCoA). These visualizations were created using the R vegan package (version 2.5-6) with sample distances displayed as scatterplots. Other figures were generated using the R ggplot2 package (v3.3.5).

## 3. Results

### 3.1. Effect of Management Rank on the Microbial Composition of Canned Raw Milk from Buffalo Farms

The study evaluated the management rank of three buffalo farms based on the ten-point requirements of the National Mastitis Council (NMC, USA) as well as buffalo-specific behavioral traits [5,25,26]. The scoring criteria are detailed in Tables S1 and S2, and the management ranks of the three farms were classified into three rankings. The highest score is referred to as A, followed by B and C (hereafter referred to as farms A, B, and C).

**Figure 1:**
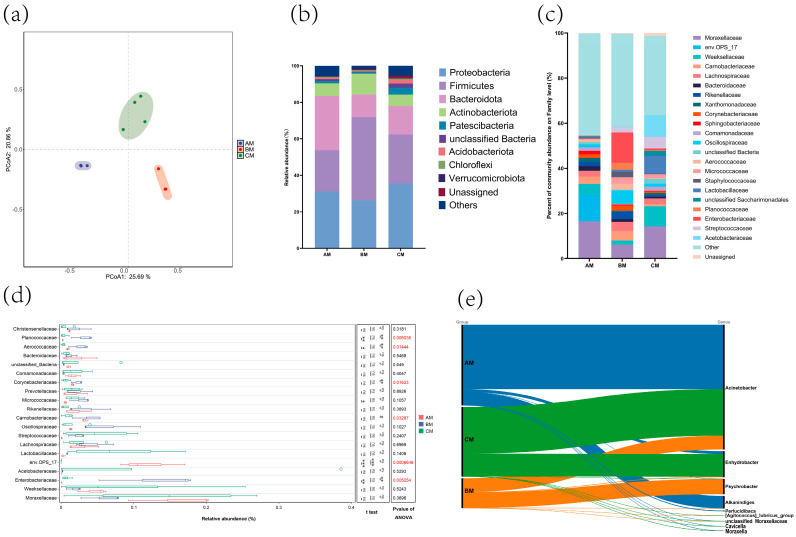
Microbial diversity profiles in raw buffalo milk. (**a**) Principal Coordinate Analysis (PCoA) of bacterial communities in raw buffalo milk from buffalo farms at different management ranks. PCoA plot with circles representing 95% confidence intervals. The relative abundances of bacterial phyla (**b**) and families (**c**) of bacterial communities in raw buffalo milk from buffalo farms at different management ranks. (**d**) Differential abundance analysis of the top 20 families revealed in raw buffalo milk at different management ranks. Multiple comparisons and ANOVA were used for statistical analysis of the data. The “ns” indicates *p* > 0.05; The “*” indicates 0.01 < *p* < 0.05; the “**” indicates 0.001 < *p* < 0.01; and the “***” indicates *p* < 0.001. (**e**) Taxonomic alluvial map of horizontal genera of the family *Moraxellaceae* in raw buffalo milk at different management ranks. AM: raw buffalo milk sample at management rank A; BM: raw buffalo milk sample at management rank B; CM: raw buffalo milk sample at management rank C.

The raw buffalo milk in these farms had an average of 73,938, 74,321, and 74,141 clean reads, respectively. Table 1 summarizes the results of the bacterial alpha-diversity indices in the milk samples. The alpha-diversity indices of bacterial communities in raw buffalo milk from farms at different management ranks showed no significant differences. These results indicate that the diversity and evenness of bacterial communities in canned raw buffalo milk from buffalo farms at different management ranks were relatively consistent. However, Principal Coordinate Analysis (PCoA) revealed significant microbial differences in sample composition among buffalo farms at different management ranks (Figure 1a). Microbial community analysis across all samples identified nine phyla considered abundant: *Proteobacteria*, *Firmicutes*, *Bacteroidota*, *Actinobacteriota*, *Patescibacteria*, unclassified *bacteria*, *Acidobacteriota*, *Chloroflexi*, and *Verrucomicrobiota*. Among these, *Proteobacteria*, *Firmicutes*, *Bacteroidota*, and *Actinobacteriota* were the most predominant phyla (Figure 1b). The most dominant families in Farm A were *Moraxellaceae* (16.5%), *env.OPS_17* (11.9%), and *Weeksellaceae* (4.6%) (Figure 1c); in Farm B, they were *Enterobacteriaceae* (13.4%), *Moraxellaceae* (6.1%), and *Oscillospiraceae* (6.9%); and the dominant families in Farm C were *Moraxellaceae* (14.3%), *Acetobacteraceae* (9.7%), and *Weeksellaceae* (8.9%).

Differential abundance analysis of the top 20 families revealed that *env.OPS_17* was significantly more abundant in Farm A than in the other farms. *Enterobacteriaceae*, *Aerococcaceae*, and *Planococcaceae* were significantly more abundant in Farm B than in the other farms, whereas *Carnobacteriaceae* and *Corynebacteriaceae* were significantly more abundant in Farm B than in Farm C (Figure 1d). The most abundant genera within *Moraxellaceae* in the farms at different management ranks were *Acinetobacter*, *Enhydrobacter*, and *Psychrobacter* (Figure 1e). Overall, while the diversity of the bacterial communities in raw milk did not show significant differences across farms with different ratings, the microbial composition of the milk was distinctly segregated based on management rank.

### 3.2. Analysis of Environmental Microbial Hazards in Buffalo Farms at Different Ranks of Management

**Figure 2:**
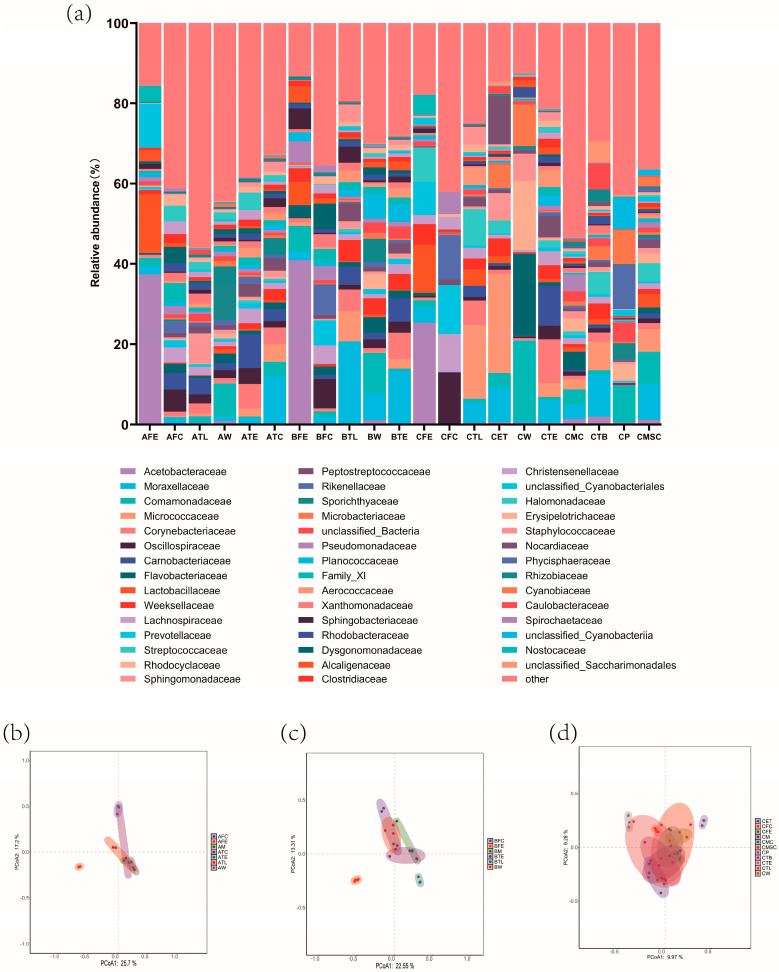
Microbiological analysis of environmental samples from buffalo pastures at different ranks of management. (**a**) Taxonomic characteristics of microbial families with relative abundance greater than 5% at the family rank in environmental samples; Principal Coordinate Analysis (PCoA) plot of milk and environmental samples; PCoA of bacterial communities in samples from buffalo farms at management ranks A (**b**), B (**c**), and C (**d**). PCoA plot with circles representing 95% confidence intervals.AFE: Feed samples from farm at management rank A; AFC: Fecal samples from farm at management rank A; ATL: Teat liner samples from farm at management rank A; AW: Drinking water samples from farm at management rank A; ATE: Teat samples from farm at management rank A; ATC: Teat dip cup samples from farm at management rank A. BFE: Feed samples from farm at management rank B; BFC: Fecal samples from farm at management rank B; BTL: Teat liner samples from farm at management rank B; BW: Drinking water samples from farm at management rank B; BTE: Teat samples from farm at management rank B. CFE: Feed samples from farm at management rank C; CFC: Fecal samples from farm at management rank C; CTL: Teat liner samples from farm at management rank C; CET: End of milking unit tube samples from farm at management rank C; CW: Drinking water samples from farm at management rank C; CTE: Teat samples from farm at management rank C; CMC: Milk canister samples from farm at management rank C; CTB: Milk transfer bucket samples from farm at management rank C; CP: Pound samples from farm at management rank C; CMSC: Muslin cloth samples from farm at management rank C.

To investigate the reasons for the microbial differences in buffalo milk from buffalo farms at varying management ranks, we performed 16S rRNA sequencing on critical control points during the milking process at these farms. The microbial families with a relative abundance greater than 5% at each risk point were analyzed. Our findings revealed there were variations in the relative abundance of bacteria at the risk points across farms with different management practices (Figure 2a).

In the feed samples, the *Acetobacteraceae* was abundantly present across farms and was the most dominant family in all cases, but its relative abundance varied between farms (37.3%, 40.9%, and 25.3%, respectively). Additionally, *Lactobacillaceae* (14.6%) and unclassified *Cyanobacteriales* (11.0%) were observed in the feed from Farm A, while *Comamonadaceae* (6.3%), *Lactobacillaceae* (5.8%), *Pseudomonadaceae* (5.4%), and *Sphingobacteriaceae* (5.2%) were detected in the feed from Farm B. In contrast, notable taxa including *Lactobacillaceae* (12.1%), *Streptococcaceae* (8.4%), *Prevotellaceae* (8.2%), *Weeksellaceae* (5.1%), and *Nostocaceae* (5.0%) were found in the feed from Farm C.

Similarly, in fecal samples from buffalo farms at different management ranks, *Oscillospiraceae* was abundantly present across all farms, but its relative abundance varied between farms (5.5%, 7.3%, and 12.9%, respectively). Additionally, in Farm B’s fecal samples, *Rikenellaceae* (7.6%), *Dysgonomonadaceae* (6.3%), and *Prevotellaceae* (6.2%) were observed, while in Farm C, *Prevotellaceae* (12.2%), *Rikenellaceae* (11.0%), *Lachnospiraceae* (9.4%), and *Spirochaetaceae* (5.5%) were detected.

The teat liner samples showed that *Sphingomonadaceae* was the dominant family in Farm A, accounting for 7.5%. In contrast, the primary families were *Moraxellaceae* (20.3%), *Micrococcaceae* (7.4%), *Corynebacteriaceae* (5.5%), and *Weeksellaceae* (5.2%) in Farm B. The major families in the teat liners of Farm C included *Micrococcaceae* (18.3%), *Streptococcaceae* (9.1%), *Corynebacteriaceae* (6.1%), and *Moraxellaceae* (5.2%), whereas the dominant taxa at the end of the milking unit tube in Farm C were *Micrococcaceae* (24.7%), *Nocardiaceae* (12.1%), *Moraxellaceae* (8.7%), *Sphingomonadaceae* (6.7%), and *Microbacteriaceae* (5.9%).

*Comamonadaceae* was abundantly present in the drinking water, but its relative abundance varied across the farms, accounting for 13.5%, 5.7%, and 19.8%, respectively. Additionally, *Sporichthyaceae* (13.5%) were observed in Farm A. *Moraxellaceae* (6.6%), *Planococcaceae* (6.2%), and *Sporichthyaceae* (5.7%) were detected in Farm B. *Flavobacteriaceae* (20.3%), *Rhodocyclaceae* (17.3%), *Microbacteriaceae* (10.3%), and *Sphingomonadaceae* (6.8%) were detected in Farm C.

Farm A introduced a teat disinfectant bath during milking, and sampling was performed from the teat dip cup, where *Moraxellaceae* (11.7%) was the dominant microorganism.

Farm C had a unique milking process, where milk from each barn’s milking machine milk can was filtered through muslin cloth and stored in a milk transfer bucket before being collectively transferred to the milk can. Additionally, buffaloes in this farm were soaked in a pond before milking, warranting additional sampling. In the milk canister, the microbial composition was dominated by *Flavobacteriaceae* (4.5%) and *Pseudomonadaceae* (4.2%). In the muslin cloth, the dominant taxa were *Moraxellaceae* (8.9%), *Comamonadaceae* (8.0%), and *Micrococcaceae* (5.6%). In the milk transfer bucket, the major taxa included *Moraxellaceae* (10.7%), *Micrococcaceae* (7.1%), *Caulobacteraceae* (6.5%), *Streptococcaceae* (5.6%), and unclassified *Saccharimonadales* (5.3%). In the pond, the microbial composition included *Phycisphaeraceae* (11.1%), *Comamonadaceae* (9.0%), *Cyanobiaceae* (8.4%), and unclassified *Cyanobacteriia* (7.9%).

Principal Coordinate Analysis (PCoA) revealed correlations between the raw milk microbiota, the teat liner, and teat dip cup in Farm A, whereas the raw milk microbiota showed a correlation with the teat skin and feed in Farm B. In contrast, raw milk microbiota was strongly correlated with the teat liner head and feed in Farm C (Figure 2b–d).

### 3.3. Further Assessment of the Contribution of Environmental Sample Sources to the Prediction of the Microbiota of Canned Raw Buffalo Milk Based on the FEAST Traceability Model

**Figure 3:**
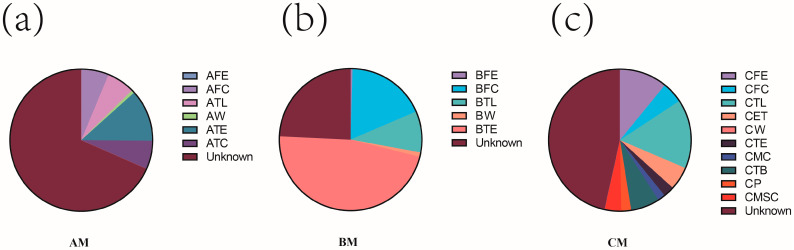
FEAST results highlight the percentage of inferred sources of contamination in buffalo milk samples from farms at management (**a**) rank A, (**b**) rank B, and (**c**) rank C. The explanations for AFE, AFC, ATL, AW, ATE, ATC, BFE, BFC, BTL, BW, BTE, CFE, CFC, CTL, CET, CW, CTE, CMC, CTB, CP, and CMSC are the same as in Figure 2.

The microbial sources in raw milk from different dairy farms were identified using the Fast Expectation-Maximization for Microbial Source Tracking (FEAST) algorithm (Figure 3a–c) [16]. The results showed that the primary sources of microbial contamination in raw buffalo milk in Farm A were the teat (12%), teat liner (6%), teat dip cup (6%), and feces (6%); while in Farm B they were the teat (47%), feces (18%), and teat liner (9%); and in Farm C they were the teat liner (16%), feed (11%), milk transfer bucket (7%), end of the milking unit tube (5%), and feces (5%).

### 3.4. Functional Differences of Microbiome in Buffalo Milk at Different Ranks of Management

**Figure 4:**
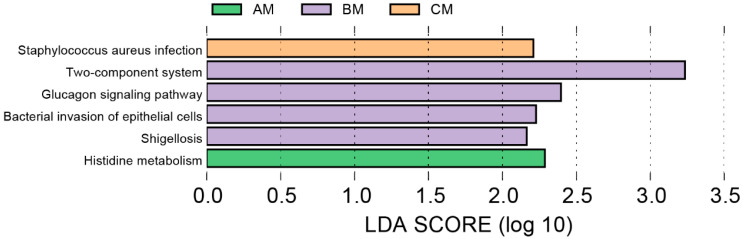
Differences in KEGG metabolic pathways in raw buffalo milk from buffalo farms at different ranks of management, analyzed using LEfSe (Linear Discriminant Analysis of Effect Size).

The PICRUSt2 [24] program was used to predict the functions of the microbial communities based on bacterial 16S rRNA sequencing data to further investigate the functional roles of bacteria in raw buffalo milk from farms at different management ranks. There were significant differences in the pathways of functional genes among the communities from different farms found by analyzing the composition of KEGG pathways and performing LEfSe differential analysis (Figure 4) [23,27]. The histidine metabolism was the identified pathway with significant difference in milk from Farm A, whereas two-component system pathways, the glucagon signaling pathway, bacterial invasion of epithelial cells, and *Shigellosis* were identified from Farm B. Meanwhile, *Staphylococcus aureus* infection was the significantly different pathway in milk from Farm C.

## 4. Discussion

The environments of buffalo farms at different management ranks significantly influenced the microbial composition of the final raw buffalo milk samples. Among these, *env.OPS_17* showed a relatively high abundance and was significantly more prevalent in Farm A compared with other farms. Existing research on *env.OPS_17* is limited; however, some studies suggest that it plays a critical role as a biofilm-forming bacterium in wastewater treatment, offering unique advantages, and is key to protein degradation under anaerobic conditions [28,29]. This study is the first to report such a high relative abundance of *env.OPS_17* in raw milk. Notably, Farm A is the only one of the three farms that employs teat disinfection before and after milking along with fully automated milking equipment, significantly reducing the influence of conventional bacterial communities on the microbial composition of the final canned raw milk. This finding lays a solid foundation for further research on *env.OPS_17*.

The relative abundances of *Enterobacteriaceae*, *Aerococcaceae*, and *Planococcaceae* were significantly higher in Farm B compared with the other farms. *Enterobacteriaceae*, in particular, not only compromises the quality of raw milk but also poses serious health risks to the animals [30]. A high prevalence of these bacteria can cause infections such as mastitis, which adversely impacts buffalo health and welfare, decreases milk production, alters milk composition, and results in economic losses for the dairy industry [31]. Interestingly, the relative abundance of *Enterobacteriaceae* in environmental samples from Farm B was low (< 0.2%), with slightly higher levels observed in the teat and feed samples (0.17% and 0.12%, respectively). However, even small amounts of *Enterobacteriaceae* during milking can serve as sources of contamination in raw milk, as noted by Mladenović et al. [32]. On the other hand, *Aerococcaceae* and *Planococcaceae* were also significantly more abundant in Farm B than in the other farms. Environmental tracing revealed that *Planococcaceae* was relatively prevalent in the drinking water and teat samples of Farm B (6.4% and 4.5%, respectively), while *Aerococcaceae* was more prominent in the teat liner and teat (2.9% and 2.3%, respectively). Previous studies have demonstrated that *Aerococcaceae* and *Planococcaceae* are commonly found in bedding materials [33], and *Aerococcaceae* has also been detected in farm air [34]. In Farm B, horizontal transmission of these bacteria likely occurred through contact with the teat, as supported by FEAST and PCoA analyses, where the teat and feed were the significant contributors to *Enterobacteriaceae* contamination. Consequently, in Farm B, it is essential to focus on mitigating horizontal transmission of these bacterial families via teats, drinking water, and milking equipment.

*Moraxellaceae* was commonly found across all farms, with no significant differences between them. However, its relative abundance in the environmental samples varied greatly. In Farm A, *Moraxellaceae* was primarily found in the teat dip cup (11.7%). In Farm B, it was widely present in the teat liner and teat (20.3% and 13.1%, respectively). In Farm C, *Moraxellaceae* was most abundant in the milk transfer bucket (10.7%), followed by the muslin cloth, end of milking unit tube, teat, and teat liner (8.9%, 8.7%, 5.2%, and 5.7%, respectively). This family was found to be widely present in raw buffalo milk [35]. The literature suggests that *Moraxellaceae* primarily colonizes mucous membranes, the skin of humans and animals, the air in livestock facilities, bedding, and other substrates [36,37]. Environmental studies also indicate that *Moraxellaceae* in raw milk may originate from feces [9,38]. Data from this study indicate that the *Moraxellaceae* found in raw buffalo milk primarily originates from post-milking stages, such as teat liners and teats. Within this family, the main contributing genus was *Acinetobacter*, a psychrotrophic bacterium that can grow during refrigerated milk storage and produce heat-resistant proteases. These proteases hydrolyze casein micelles, affecting milk quality and potentially causing spoilage in downstream products such as cheese and yogurt [39]. Therefore, cleaning teat liners and teats can significantly reduce the horizontal transmission of psychrotrophic bacteria like *Acinetobacter* within *Moraxellaceae*, thereby minimizing their impact on the safety and quality of the final product.

*Enterobacteriaceae*, which was widespread in Farm B, had associated dominant metabolic pathways (e.g., *Shigellosis*) that are directly related to foodborne illnesses [40]. Improper processing could lead to poisoning or other health issues for consumers [41]. *Staphylococcus aureus* infection pathways had higher contributions, although this bacterium was less frequently detected in raw milk. Our study indicates that in Farm C, *Staphylococcaceae* was widely present in the teat liner and teat (4.4% and 2.1%, respectively). *Staphylococcaceae* can enter the intramammary area either through progressive colonization from the teat apex or by being propelled into the intramammary area during vacuum fluctuations of the milking machine, thereby inducing bovine mastitis [42]. Their presence on milking equipment and animal contact points highlights potential weaknesses in hygiene protocols. These findings align with previous research indicating that enhanced cleaning and sanitization of milking equipment and teats can significantly reduce microbial contamination, improving milk quality and safety [43].

Generally, this study reveals that the management practices and environmental conditions of buffalo farms significantly influence the microbial composition of raw milk. The impact of the teat and teat liners on the microbial composition of the final raw milk should be a focus for all farms, as these areas are often overlooked in daily farm cleaning routines; specific risks should also be addressed depending on the farm’s management rank, including the influence of teat dip cups, feces, and feed. These results underscore the importance of tailored hygiene protocols and management strategies to mitigate contamination and enhance the safety of raw milk products, which can be practically applied to develop targeted interventions that improve milk quality and ensure compliance with food safety standards. To demonstrate the microbial communities at the identified milking risk points and how these bacteria affect milk quality, further research and experimental validation are necessary.

## 5. Conclusions

This study analyzed the microbiota characteristics and sources of contamination in canned raw buffalo milk from three buffalo farms with different management levels in Guangxi Province, China. The results showed significant differences between the milk and environmental microbiota between the farms with different management levels. Fast Expectation-Maximization for Microbial Source Tracking indicated that the teat was the primary source of milk contamination in Farms A and B. On the other hand, the main sources in Farm C were teat liners and feed. Furthermore, the transmission routes of specific bacterial families, such as *Moraxellaceae* and *Enterobacteriaceae*, varied between the farms. This study provides a foundation for developing targeted interventions to improve milk quality, exploring the relationship between buffalo health and milk microbiota as well as identifying biomarkers for the early detection of contamination or disease.

## Figures and Tables

**Table 1:** Comparison of alpha-diversity indices in raw buffalo milk samples from farms at different ranks of management.

Sample	AM	BM	CM	*p* Value
ASVs	1528.3333 ± 185.4730	1782.3333 ± 123.8287	1859.2500 ± 1400.1788	0.918
ACE	1531.0378 ± 185.1027	1788.7559 ± 123.6023	1862.6068 ± 1398.7170	0.918
Chao1	1529.3634 ± 185.5027	1784.2837 ± 123.4209	1860.0646 ± 1399.7299	0.918
Simpson	0.9761 ± 0.0107	0.9753 ± 0.0135	0.9408 ± 0.0575	0.518
Shannon	7.9736 ± 0.4333	8.4521 ± 0.5869	7.2540 ± 2.1636	0.661
Good coverage	0.9998 ± 0.000	0.9997 ± 0.0000	0.9998 ± 0.0001	0.098

Note: All data shown were (mean ± standard deviation). The *p* value was obtained with one-way ANOVA.

## Data Availability

The original contributions presented in this study are included in the article/Supplementary Materials. Further inquiries can be directed to the corresponding authors.

## Supplementary Materials

The following supporting information can be downloaded at: https://www.mdpi.com/article/10.3390/foods13244080/s1, Table S1: Buffalo ranch management rank rating criteria. Table S2: Buffalo farm management scoring summary and rating evaluation.
