# Supplementary material for: The Microbial Diversity and Traceability Analysis of Raw Milk from Buffalo Farms at Different Management Ranks in Guangxi Province
Source: Foods. 2024 Dec 17;13(24):4080. doi: 10.3390/foods13244080 (PMC11675397; doi:10.3390/foods13244080)
Supplement: Supplementary file 1 [file foods-13-04080-s001.zip › foods-3368674-supplementary.pdf]

Table S1: Buffalo Ranch Management Rank Rating Criteria.

Management measure description	Scoring criteria
1. Keep water buffalo standing for at least 30 minutes after milking	5 points: All water buffalo can stand for at least 30 minutes after milking.<br>4 points: Most water buffalo can stand for 30 minutes, but a few stand for shorter period.<br>3 points: Most water buffalo can stand for about 20 minutes.<br>2 points: A few water buffalo can stand for 20 minutes, while others stand for shorter periods.<br>1 point: Only a few water buffalo can stand for a few minutes.<br>0 points: No measures, water buffalo lie down immediately after milking.
2. Reduce the number of water buffalo to prevent overcrowding	5 points: The number of water buffalo is well-controlled, and there is no overcrowding.<br>4 points: The number of water buffalo is reasonable, with occasional mild overcrowding.<br>3 points: The herd is often slightly overcrowded but still maintains healthy standards.<br>2 points: The herd is too large, and significant overcrowding is present.<br>1 point: Severe overcrowding, affecting buffalo activity and health.<br>0 points: Severe overcrowding, leading to significant declines in health and productivity.
3. Clean the barn twice a day and maintain clean bedding	5 points: The barn is thoroughly cleaned twice a day, and bedding is kept clean and sufficient.<br>4 points: The barn is cleaned twice a day, but the bedding occasionally has slight dirt.<br>3 points: Occasionally, cleaning is inadequate, and some bedding areas are dirty.<br>2 points: Cleaning is done only once a day, and bedding is often dirty.<br>1 point: The bedding and barn are not cleaned regularly, resulting in a dirty environment.<br>0 points: Rarely cleaned, and bedding is never replaced.
4. Manually clean the milking area twice a day	5 points: The milking area is manually cleaned twice a day, and the environment is clean with no dirt.<br>4 points: The area is cleaned twice a day, but sometimes parts are not thoroughly cleaned.<br>3 points: The area is cleaned once a day, and some parts are messy.<br>2 points: Cleaning is irregular, with obvious hygiene issues.<br>1 point: Cleaning is done occasionally, and the environment is poor.<br>0 points: The farm is almost never cleaned, with a poor environment.
5. Milk buffalo with clinical symptoms last	5 points: Strictly enforced, all buffalo with clinical symptoms are milked last.<br>4 points: Most buffalo with clinical symptoms are milked last, with occasional neglect.<br>3 points: Sometimes not strictly enforced, and some buffalo with clinical symptoms are not milked last.<br>2 points: Often not enforced, and several symptomatic buffalo are milked out of order.<br>1 point: Almost never enforced, all buffalo are milked out of order.<br>0 points: No measures taken.
6. Milk buffalo with elevated somatic cell counts (>250,000 cells/mL) last	5 points: Fully enforced, all buffalo with elevated somatic cell counts are milked last.<br>4 points: Most buffalo with elevated somatic cell counts are milked last, with occasional neglect.<br>3 points: Sometimes not strictly enforced, and some buffalo with elevated somatic cell counts are not milked last.<br>2 points: Often not enforced, and most buffalo with high somatic cell counts are milked out of order.<br>1 point: Almost never enforced, all buffalo are milked out of order.<br>0 points: No measures taken.

7. All water buffalo udders are cleaned using disinfected towels	5 points: Strictly enforced, each buffalo uses a disinfected towel.<br>4 points: Most buffalo use disinfected towels, but occasionally one towel is shared.<br>3 points: Some buffalo use disinfected towels, but most share one.<br>2 points: Towel management is chaotic, and many buffalo share a disinfected towel.<br>1 point: Hardly any buffalo are given individual disinfected towels.<br>0 points: No cleaning measures taken.
8. Dirty udders are cleaned with water and dried	5 points: Strictly enforced, all dirty udders are cleaned and dried before milking.<br>4 points: Most dirty udders are cleaned and dried, with occasional neglect.<br>3 points: Cleaning is inadequate, and some dirty udders are not handled in time.<br>2 points: Cleaning is insufficient, and many dirty udders are neglected.<br>1 point: Cleaning is not frequent, and udders are generally dirty.<br>0 points: No cleaning measures taken.
9. All water buffalo undergo pre-milking stripping	5 points: All buffalo undergo pre-milking stripping.<br>4 points: Most buffalo undergo pre-milking stripping, with occasional neglect.<br>3 points: Pre-milking stripping is inconsistent, and some buffalo do not undergo it.<br>2 points: Pre-milking stripping is done casually, and the execution is not standardized.<br>1 point: Hardly any buffalo undergo pre-milking stripping.<br>0 points: No pre-milking measures taken.
10. Milkers wear gloves during milking	5 points: All milkers wear gloves every time they milk.<br>4 points: Most milkers wear gloves, with occasional neglect.<br>3 points: Some milkers wear gloves, but the practice is not strict.<br>2 points: Gloves are rarely worn.<br>1 point: Milkers almost never wear gloves.<br>0 points: No gloves worn.
11. Pre-milking disinfectant bath	5 points: Strictly enforced, all buffalo are treated with disinfectant before milking.<br>4 points: Most buffalo are disinfected, with occasional neglect.<br>3 points: Some buffalo receive disinfectant treatment, but quality is average.<br>2 points: Disinfection is infrequent and of low quality.<br>1 point: Hardly any disinfecting is done.<br>0 points: No disinfection measures taken.
12. Post-milking treatment with medicated bath cups	5 points: Strictly enforced, all buffalo use medicated bath cups after milking.<br>4 points: Most buffalo use medicated bath cups, with occasional neglect.<br>3 points: Some buffalo use medicated bath cups, but the disinfectant quality is average.<br>2 points: Medicated bath cups are used infrequently, and the quality is poor.<br>1 point: Almost no post-milking treatment is done.<br>0 points: No post-milking treatment.
13. Milking equipment is cleaned with hot water after milking buffalo with clinical or subclinical mastitis	5 points: Strictly enforced, all milking equipment is cleaned with hot water after milking symptomatic buffalo.<br>4 points: Most symptomatic buffalo's milking equipment is cleaned, but occasional neglect occurs.<br>3 points: Some symptomatic buffalo's equipment is not cleaned after milking.<br>2 points: Cleaning is inconsistent, and equipment is not always clean.<br>1 point: Milking equipment is rarely cleaned.<br>0 points: No cleaning measures taken.

14. Milking cups are replaced according to manufacturer standards	5 points: Strictly follows the manufacturer's recommendation for regular replacement.<br>4 points: Generally follows the standard, with occasional delays.<br>3 points: Replacements occur less frequently than recommended, but are acceptable.<br>2 points: Replacements are not timely, and milking cups are overused.<br>1 point: Milking cups are rarely replaced.<br>0 points: Milking cups are almost never replaced.
15. Work with a veterinarian to develop treatment plans and evaluate monthly	5 points: Close cooperation with a veterinarian, and treatment plans are updated and evaluated monthly.<br>4 points: Cooperation is present, but the evaluation is slightly inadequate.<br>3 points: Treatment plans are created but evaluated infrequently.<br>2 points: Treatment plans are outdated, with a lack of evaluations.<br>1 point: Hardly any effective treatment plans are in place.<br>0 points: No treatment plan.
16. Use selective dry cow therapy (SDCT) during the dry period	5 points: The farm has a complete SDCT plan in place.<br>4 points: The farm has SDCT, but it is not strictly followed.<br>3 points: The farm uses blanket dry cow therapy (BDCT) instead of SDCT.<br>2 points: The farm has BDCT, but it is not strictly followed.<br>1 point: Antibiotics are rarely used during the dry period.<br>0 points: No antibiotics are used.
17. Add appropriate minerals to feed	5 points: Proper minerals are added to the dry feed.<br>4 points: Most buffalo receive minerals, but occasionally some may lack them.<br>3 points: Some buffalo are missing minerals in their feed.<br>2 points: Mineral addition is poorly managed, and many buffalo are undernourished.<br>1 point: Rarely are minerals added.<br>0 points: No minerals are added.
18. Optimize feed based on the farm's specific needs	5 points: Feed is fully optimized according to the farm's needs, meeting the best nutritional standards.<br>4 points: Feed mostly meets the needs, with occasional minor deficiencies.<br>3 points: Feed is partially optimized but lacks refinement.<br>2 points: Feed management is poor, and nutritional content is insufficient.<br>1 point: Feed management is chaotic, seriously affecting nutrition.<br>0 points: No feed optimization.
19. Regular cleaning and disinfection of milking equipment	5 points: Milking equipment is cleaned and disinfected according to the highest standards.<br>4 points: Regular cleaning and disinfection, with occasional neglect.<br>3 points: Cleaning occurs, but the process could be more thorough.<br>2 points: Cleaning occurs, but inadequately or irregularly.<br>1 point: Milking equipment is rarely cleaned.<br>0 points: Milking equipment is never cleaned.
20. Control environmental conditions such as temperature and humidity	5 points: Environmental conditions are controlled strictly to maintain optimal temperature and humidity levels.<br>4 points: The environmental conditions are usually controlled, with occasional neglect.<br>3 points: Environmental control is average and sometimes fluctuates.<br>2 points: Environmental conditions are not effectively controlled.<br>1 point: Environmental management is poor, affecting buffalo health.<br>0 points: No environmental control.

Final Score Rating	90-100: Excellent management level, with almost all measures strictly implemented. (A)<br>70-89: Good management level, with room for improvement. (B)<br>40-69: Average management level, with significant room for improvement. (C)<br>0-39: Poor management level, with almost no effective measures implemented. (D)

Table S2: Buffalo farm management scoring summary and rating evaluation

Scoring Item	Buffalo Ranch 1	Buffalo Ranch 2	Buffalo Ranch 3
1	5	5	5
2	5	5	5
3	3	0	3
4	5	2	5
5	5	5	5
6	4	4	5
7	3	1	5
8	3	0	5
9	4	0	5
10	3	0	5
11	0	0	5
12	0	0	5
13	5	5	5
14	1	0	3
15	5	5	5
16	2	0	5
17	5	4	5
18	4	3	4
19	4	2	5
20	5	5	5
Total	71	46	95
Rating	B	C	A
